# Long term outcomes from the early days of minimally invasive thymoma surgery for Myasthenia Gravis patients: a follow-up of 19 years

**DOI:** 10.3389/fsurg.2024.1486282

**Published:** 2024-11-12

**Authors:** Erkan Kaba, Berker Özkan, Jahnavi Kakuturu, Eyüp Halit Yardımcı, Eren Erdoğdu, Berk Çimenoğlu, Salih Duman, Alper Toker

**Affiliations:** ^1^ Department of Thoracic Surgery, School of Medicine, Demiroglu Bilim University, İstanbul, Türkiye; ^2^ Department of Thoracic Surgery, Istanbul Medical School, Istanbul University, İstanbul, Türkiye; ^3^ Department of Cardiovascular Surgery, School of Medicine, West Virginia University, Morgantown, WV, United States; ^4^ Department of Thoracic Surgery, Group Florence Nightingale Hospitals, İstanbul, Türkiye

**Keywords:** thymoma, Myasthenia Gravis, VATS, follow-up, recurrence

## Abstract

**Background:**

Long-term survival outcomes are crucial for accurately determining the effectiveness of treatment in an indolent disease like thymoma. We aimed to analyze the clinical findings in terms of survival and relapse patterns with a median follow up of 105 months (8.7 years) in patients with thymoma and myasthenia gravis who underwent minimally invasive surgery between 2002 and 2015.

**Methods:**

A total of 59 pathologically confirmed Masaoka Stage I and II thymoma patients with Myasthenia Gravis (MG) who underwent minimally invasive thymoma resection were included in this study. Primary aim of this study is to evaluate recurrences, overall and disease free survival in patients with a thymoma and MG in the long run. We also aimed to study the changes in Myasthenia Gravis medication during the follow-up.

**Results:**

The mean age of the patients was 47.6 +/13.5 years. Seventeen patients were at Masaoka Stage I and 42 were at Masaoka Stage II. The median follow-up time was 105 months. The mean size of the tumor was 3.6 cm +/16.2 cm. Twenty-one patients (35%) received adjuvant radiotherapy. There was no postoperative mortality and median length of hospital stay was 4 days. Two patients developed recurrences both presented with pleural metastases. Eight patients died because of non-oncologic pathologies. 10-year disease free survival and overall survival rates were calculated to 96.6% and 86.4% respectively.

**Conclusion:**

The 10-year survival analysis and current myasthenic status of stage I-II thymoma patients with myasthenia gravis who underwent minimally invasive surgery demonstrate that these procedures are both safe and effective.

## Introduction

Complete resection is the standard treatment in Masaoka stage I and II thymomas, provided all oncological principles of surgery are followed. Long term survival has always been the center of debate regarding thymoma resections with minimally invasive surgical techniques. In the literature, long term results of minimally invasive surgeries have been reported to have a 1 year minimum follow up with a median of 30 to 58 months of survival in the longest follow ups (1–5). There has always been a concern for increased risk of local recurrence with time, because of the reduced safety margins and the possible rupture of the capsule causing implantation of the tumor during endoscopic manipulations (6). Recurrence due to minor implantation in an indolent tumor would be expected to occur later in the life. For this reason, longer follow up is required to validate minimally invasive surgery for thymoma resection.

## Methods

### Patient selection and preoperative work up

A total of 59 patients who underwent thymoma resection with minimally invasive techniques between September 2002 and March 2015, were evaluated retrospectively. Inclusion criteria was specific to patients with pathologically confirmed Masaoka stage I-II thymomas and a diagnosis of myasthenia gravis (MG). All procedures were performed by the same surgical team at Istanbul University, Istanbul Faculty of Medicine, Department of Thoracic Surgery and Demiroglu Bilim University, Group Florence Nightingale Hospitals, Department of Thoracic Surgery.

We planned to restrict the study to the MG population, so as to keep the group uniform. All cases were diagnosed with MG preoperatively, and all patients’ thymomas were found during the work up in the neurology clinics. The patients underwent re-assessment by the neurology departments of corresponding hospitals in one week before the surgery (Istanbul Medical School, Department of Neurology and Demiroglu Bilim University, Group Florence Nightingale Hospitals, Department of Neurology). All patients had Acetylcholine antibody receptor levels and single fiber electromyography and chest computerized tomography (CT). All patients were evaluated by an experienced anesthesiologist preoperatively. If the anesthesiologist had concerns for bulbar muscle weakness, then the patients were reassessed by a neurologist. Additionally, the necessity for post-operative intensive care unit (ICU) admission was based on the anesthesiologist's and neurologist's recommendations.

The decision to proceed with operative intervention was based on detection of thymoma in radiological examinations without considering the clinical stage of myasthenia gravis. For all cases of thymoma, radiological evaluation was facilitated using CT chest without a contrast as all the patients had MG. After 2007, full body fluorodeoxyglucose positron emission computed tomography (FDG-PET CT) evaluation was performed in addition to CT chest. The aim of performing a PET CT was for differential diagnosis and staging (particularly mediastinal lymph node evaluation and detection of possible pleural implants). In cases where local invasion was suspected, contrast-enhanced magnetic resonance imaging (MRI) of the chest was performed. None of the cases were biopsied preoperatively. Neoadjuvant therapy was not given to any of the cases in this study. Patients who had intravenous immunoglobulin (IVIG) therapy underwent treatment 5–7 days prior to surgery. Surgery was always performed as the first case of the day. Patients received their medications as usual prior to the surgery with a small sip of water and follow-up treatments were maintained as they are scheduled.

All patient data was prospectively collected, and early patient data was submitted to International Thymic Malignancies Interest Group Database. Follow ups were done annually with a CT scan and last follow ups before this study was performed with phone calls by the authors.

### Surgical details and postoperative stay

**Figure 1:**
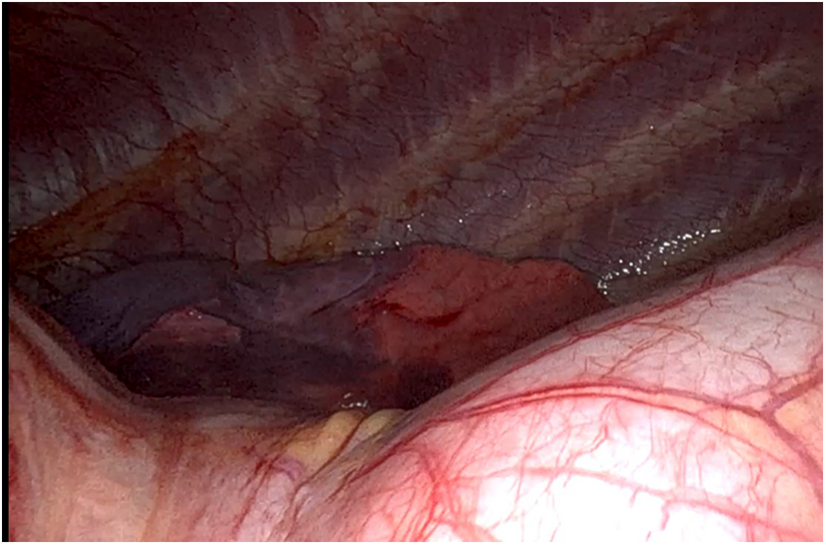
Intraoperative left phrenic nerve.

**Figure 2:**
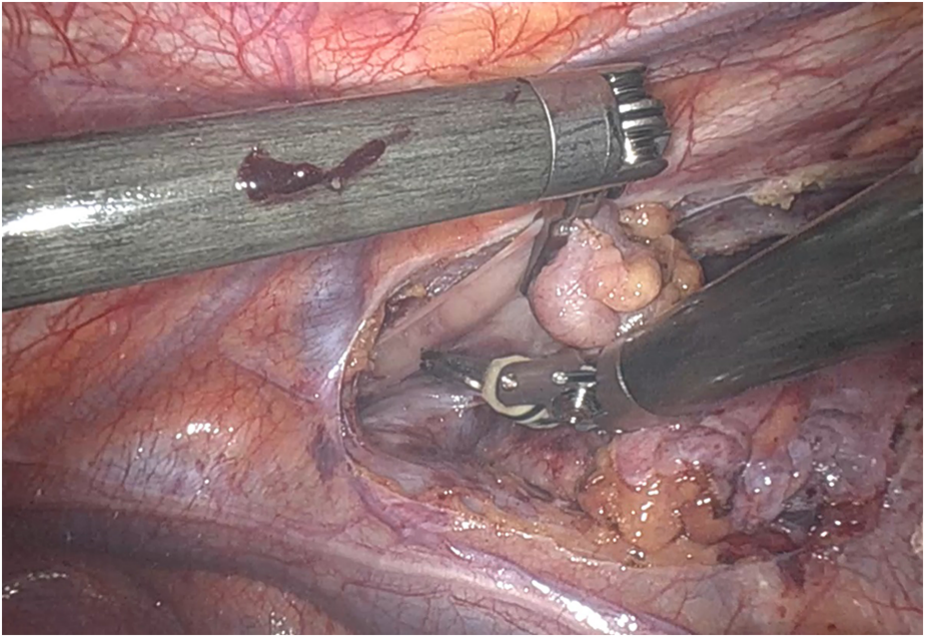
Intraoperative right thymic pole pull-down.

**Figure 3:**
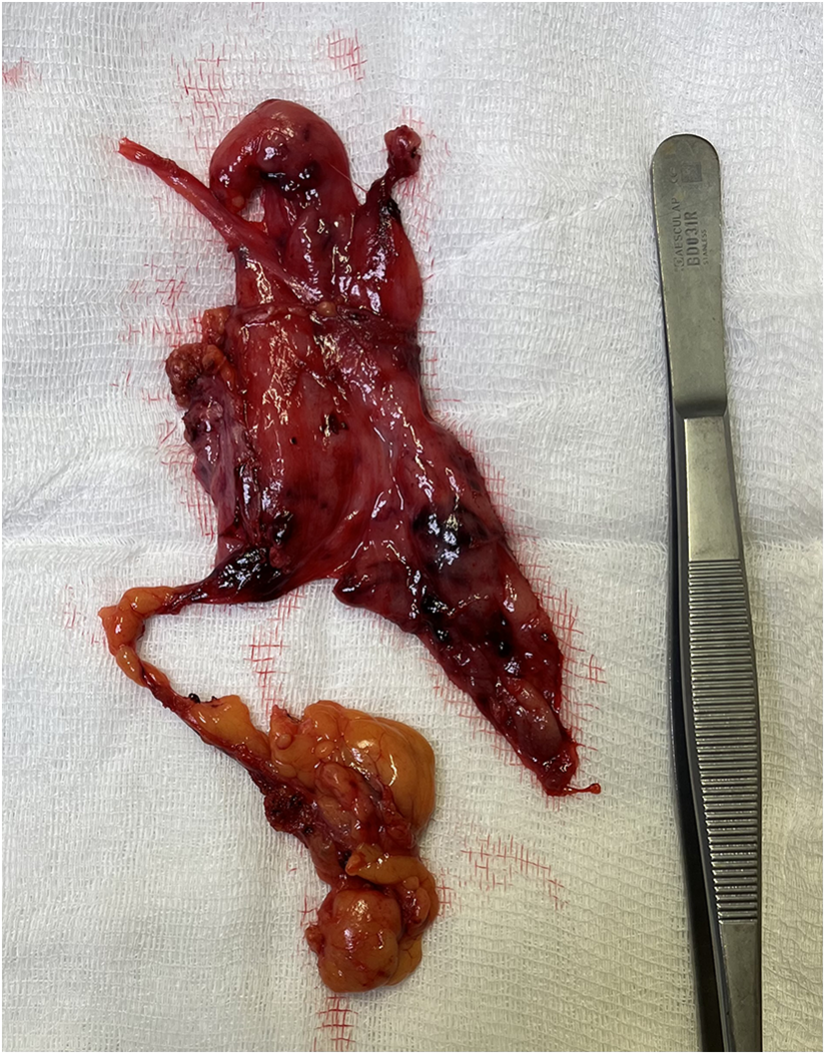
Thymectomy specimen.

All cases were operated completely with minimally invasive techniques, 58 patients underwent VATS resection by a right sided approach, while 1 patient underwent a robotic right assisted approach. Extended thymectomy with “en bloc” resection was performed, including removal of the contiguous right and left mediastinal pleura, mediastinal and cardiophrenic fatty tissues, and dissection of aorta-pulmonary window in addition to complete thymectomy. After the year 2010, the surgery included mediastinal lymph node sampling. Resection principles included the “no touch” technique, which prohibits direct grasping of the thymoma tissue as previously described by ITMIG (6). The VATS technique employed three right sided ports. At the onset of surgery, if the thymoma shows macroscopic adhesions to the lung, a wedge resection is performed on the adjacent lung tissue and included in the thymoma specimen. Similarly, if adhesions are present in the pericardial plane, the pericardium is opened, and the adhered area is incorporated into the specimen. If the phrenic nerve appears to be invaded, it is sacrificed, and diaphragm plication is performed during the same procedure. Dissection was begun with normal thymic tissue, from either the upper pole or the lower pole on the right side. In thymoma surgery, particularly in myasthenic cases, meticulous care should be taken to visualize and preserve the left phrenic nerve (Figure 1). We recommend employing the “pull-down” technique for the complete removal of the thymus's upper poles (Figure 2). If the upper poles do not come off easily during traction, it suggests that the surrounding connective tissue is being pulled along with the thymic tissue. Ensuring dissection within the correct anatomical plane is crucial to avoid potential technical complications. The thymoma was mobilized as the last part of the operation, and the specimen was always removed in a retrieval bag (Figure 3). One of the incisions was enlarged for retrieval of the thymoma by exercising extreme caution to prevent specimen disruption. All pathological specimens were anatomically orientated in the pathology laboratory, and this was facilitated by the primary surgeon or the first assistance of the case. Tumors with mediastinal pleural invasion, which may be misdiagnosed as positive surgical margins by the pathologist, were oriented as the pleura not being a surgical margin, and negative surgical margins were decided with the pathologist to avoid any misunderstanding.

All cases had a single right sided chest tube, or a Jackson Pratt drainage catheter, or both after surgery. Since all cases were in patients with MG, the need for postoperative ICU monitoring was decided with the attending anesthesiologists individually at the end of each surgery. Preoperative drug therapy for MG was continued postoperatively according to each individual's routine regimen until the first outpatient neurology assessment. Patients were usually discharged from the hospital the day after the removal of the chest tube.

### Follow-up period and adjuvant therapy

The decision to proceed with surgery and the need for postoperative oncological treatment was made by the multidisciplinary tumor board including thoracic surgery, radiology, nuclear medicine, radiation and medical oncology, and pathology. Postoperative routine follow-ups were facilitated by both neurology and thoracic surgery. Radiological follow-up consisted of chest CT evaluation at 3 months, 1 year, and annually for 10 years, followed by every other year, thereafter. All cases that were pathologically diagnosed with capsular invasion prior to 2010, received adjuvant radiotherapy (RT). After 2010, only the cases diagnosed with WHO types B2 and B3 having capsular invasion received adjuvant RT. Postoperative PET CT evaluation was performed for persistent pleural effusion, pleural thickening, solid lesions seen in the operative bed or adjacent mediastinal and lung parenchymal tissue. Surgical excision was preferred in suspicious cases of radiological recurrence.

Cases were analyzed according to their age, gender, symptoms, myasthenic status, medical therapy prescribed for MG, adjuvant therapy, tumor size, lymph node dissection status, WHO pathological thymoma classification, recurrence rate, disease free survival (DFS), and 10-year overall survival (OS) results. Number Cruncher Statistical System (NCCS) Statistical Software (Utah, USA) program was used for statistical analysis. Kaplan-Meier survival analysis was used for survival evaluation. Statistical significance was set at *p* < 0.05.

## Results

Mean age of the cohort was 47.6 ± 13.5 years (range 21–75 years) and 54.2% were female. With regard to the Masaoka Stage, 17 cases (28.8%) were Stage I and 42 cases (71.2%) were Stage II. Mean tumor size was 36.7 ± 16.2 mm (range 7–80 mm). Myasthenic status conforming to the Osserman-Genkins classification was observed as follows: 15 cases (25.5%) were Stage I, 22 cases (37.3%) Stage IIA, 14 cases (23.7%) Stage IIB, 3 cases (5.0%) Stage IIIA, and 5 cases (8.5%) Stage IIIB. Forty-two (71.2%) cases were evaluated radiologically with PET CT preoperatively.

Twenty-seven patients (45.8%) received preoperative IVIG therapy, and 3 (11.1%) of whom required intermittent IVIG therapy after the operation. Fifty-four cases (91.5%) were found to be on pyridostigmine therapy preoperatively and 38 cases (70.4%) no longer required pyridostigmine therapy after the operation. Thirty patients (50.8%) were prescribed steroids preoperatively, 22 (73.3%) of whom did not use steroids in the postoperative period. Two patients who were not on steroids before the operation were prescribed steroid therapy postoperatively. Five patients (8.5%) were receiving azathioprine preoperatively; 4 (80%) of these patients continued the therapy after surgery. Nine patients who were not on any immunosuppressive therapy before the operation were started on therapy in the follow-up period.

Only 1 case of thymoma resection was performed with robotic surgery (via right chest). All other cases were operated on using a right sided VATS approach with 3 ports. There were not any major surgical complications or unexpected conversions during the surgeries. Seventeen patients (28.8%) underwent additional mediastinal lymph node dissection during surgery. Pathologically, 18 cases (30.5%) were found to have type A-AB, 14 cases (23.7%) type B1, 22 cases (37.3%) type B2, and 5 cases (8.5%) type B3 according to the WHO pathologic classification of thymoma. ICU admission was needed for 33.9% of cases postoperatively. Median ICU stay length was 1 day (0–52 days) and median hospital stay was 4 days (2–68 days). Twenty-one cases (35.6%) had adjuvant RT.

**Figure 4:**
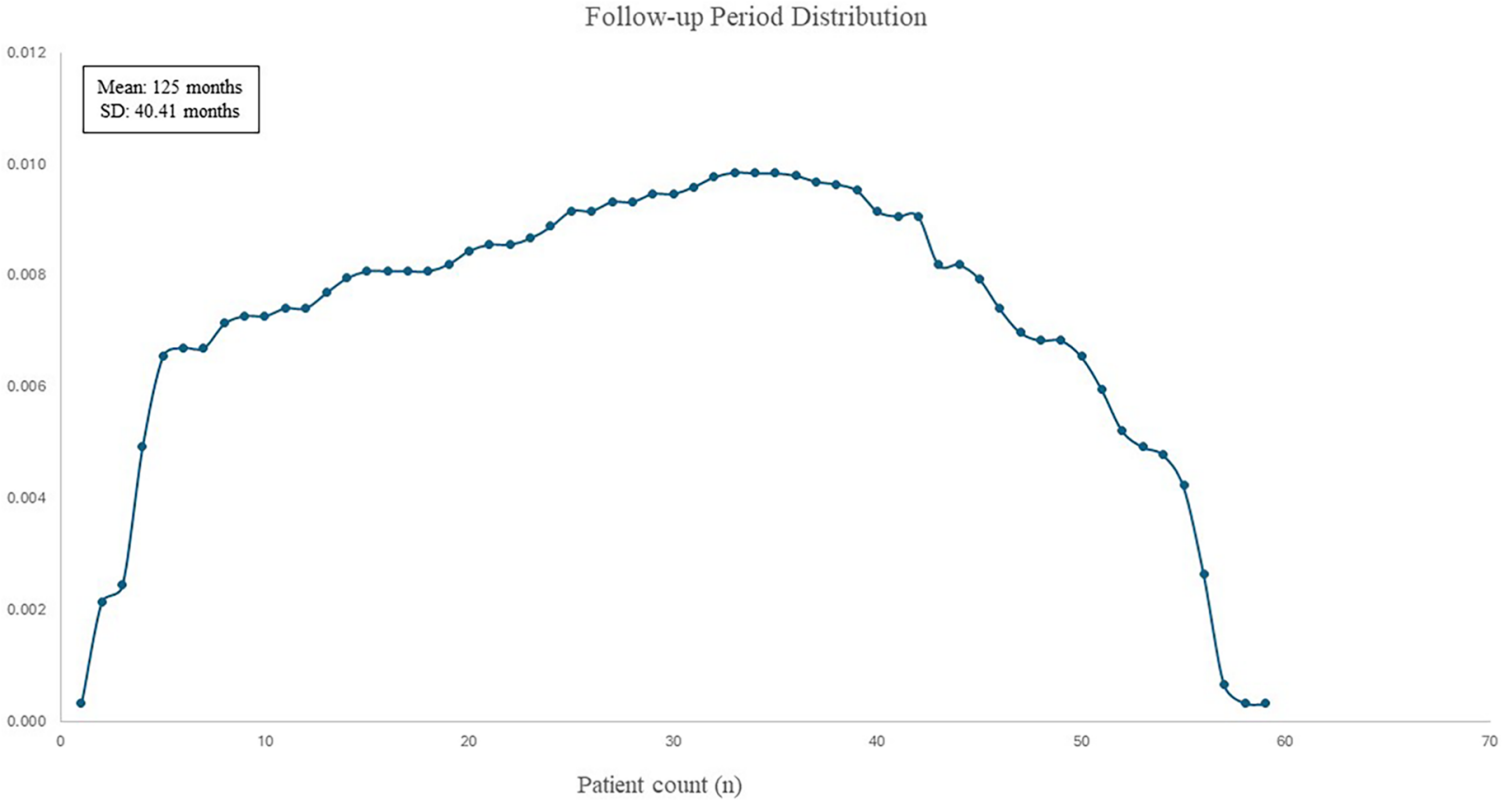
Distribution curve of patients’ follow-up period.

**Figure 5:**
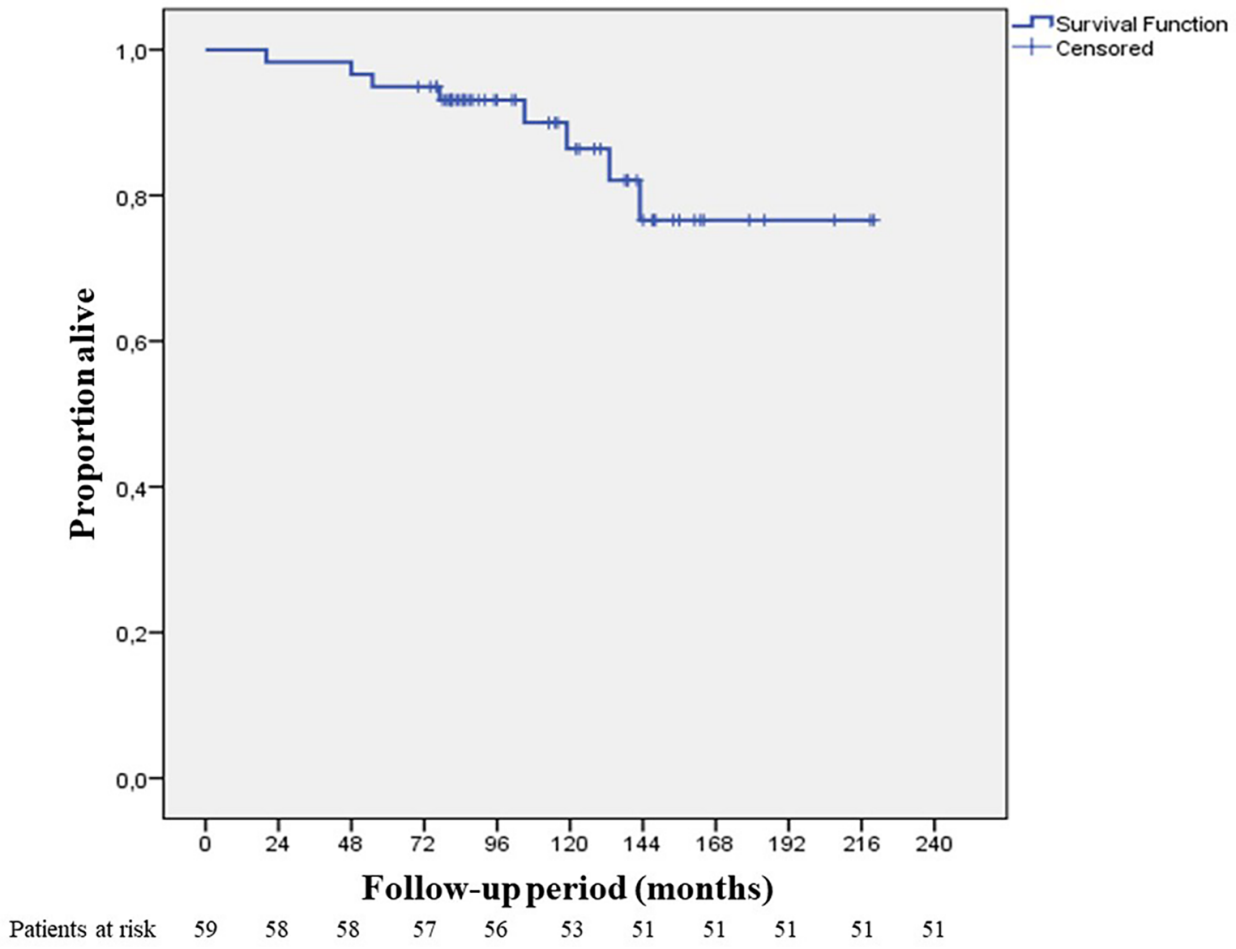
10-year overall survival.

Median follow-up period was 105 months (20–220 months) (Figure 4). In the 10-year follow-up period, oncological mortality secondary to thymoma was not observed. During the follow-up period, there were 8 cases of mortality. Overall 10-year survival was 86.4% (mean 193.11 months, SD: 8.66 months) (Figure 5).

**Figure 6:**
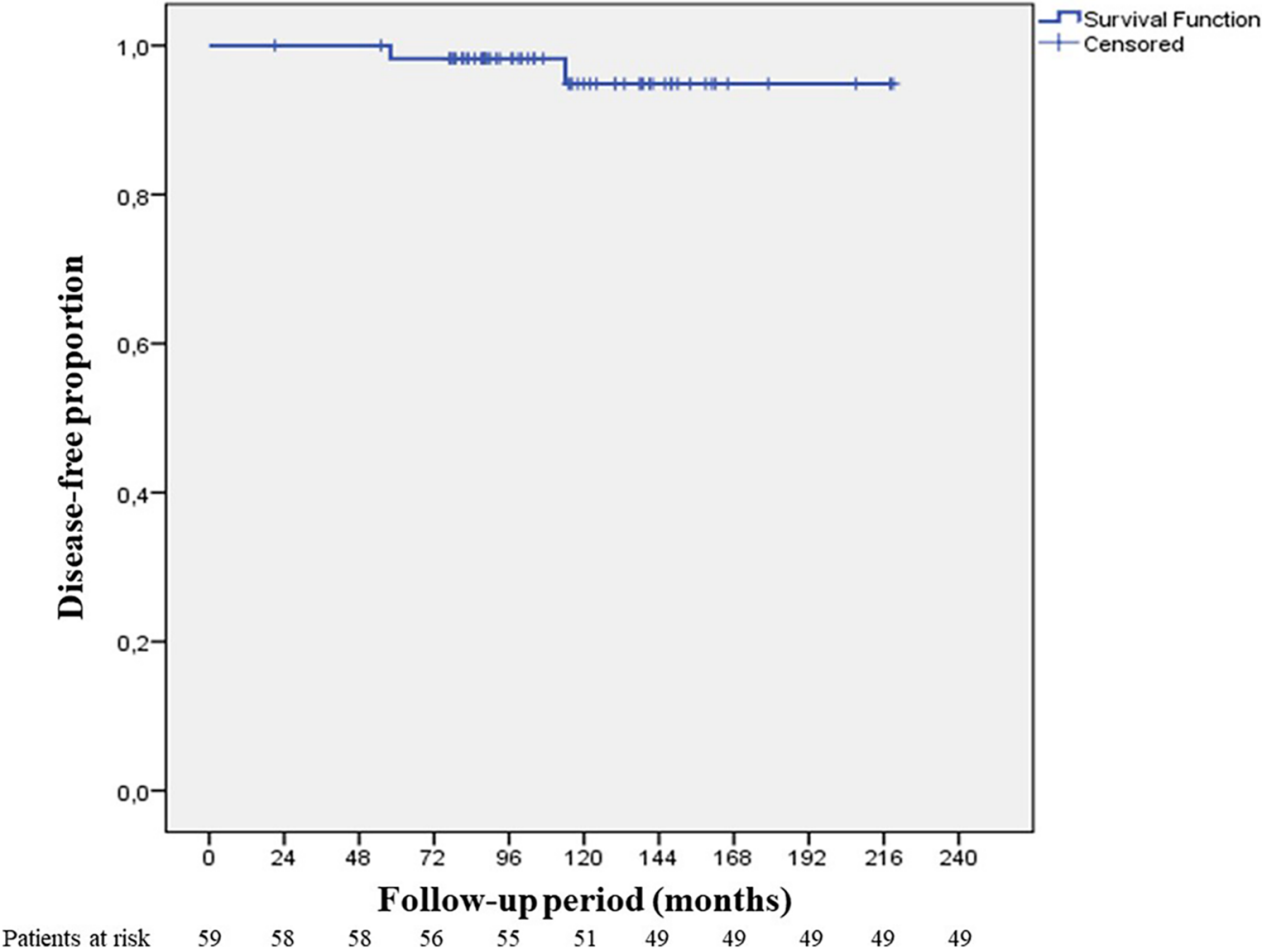
10-year disease free survival.

Disease-free survival at 10 years was found to be 96.6% (median 104 months) (Figure 6). Recurrence was observed in 2 cases (3.4%) both of which were Masaoka Stage II. One patient had recurrence at the 58th month and was WHO type B3 case who had received adjuvant RT. On the other hand, the second patient had recurrence at the 114th month, and was WHO type B1 having received no adjuvant therapy. Recurrence developed in pleural and lung parenchymal tissue in one patient for which pleurectomy, decortication, lung wedge resections, and diaphragmatic resection was performed, followed by adjuvant chemotherapy. She was subsequently followed up for 3 years without recurrence. The other patient only had one pleural metastatic deposit which was excised with pleurectomy. Additionally, 3 cases underwent reoperation with open surgery due to concern of local recurrence within 2 years of postoperative period. All these 3 cases had adjuvant RT. Middle lobectomy was performed in one case and wedge resections were performed for the other two cases. Pathological examination did not report thymoma recurrence and confirmed radiation fibrosis.

## Discussion

In this study, we have reported on Masaoka Stage I and Stage II thymoma patients with myasthenia Gravis who were operated with minimally invasive surgery and completed longitudinal follow up of 19 years. The study was restricted to the MG population for several reasons. First, MG patients are expected to develop signs and symptoms of recurrence earlier than non-MG patients. Second, there remains discussion on whether there is less benefit following thymectomy in patients with MG and we hoped to contribute some solid data to this unresolved issue. Third, we sought to understand if there is potentially more benefit to MG patients when follow up time is longer.

Oncologic outcomes following VATS and robotic assisted thymoma surgery have been studied in the literature several times with varying follow up periods, including early and mid-term outcomes, as well as long term outcomes (7–11). In reviewing these studies, mid-term outcomes were considered as a median follow up period of 27–48 months, whereas other publications considered long term outcomes with a median follow up of 43–59.5 months. In this study, we have reported on patients who completed a long follow up period, with a median follow up time up to 105 months. As the follow up period intersects with the start of our experience with robotic surgery for thymoma resection, this study includes only one robotic thymectomy with the remaining cases using the VATS approach.

**Figure 7:**
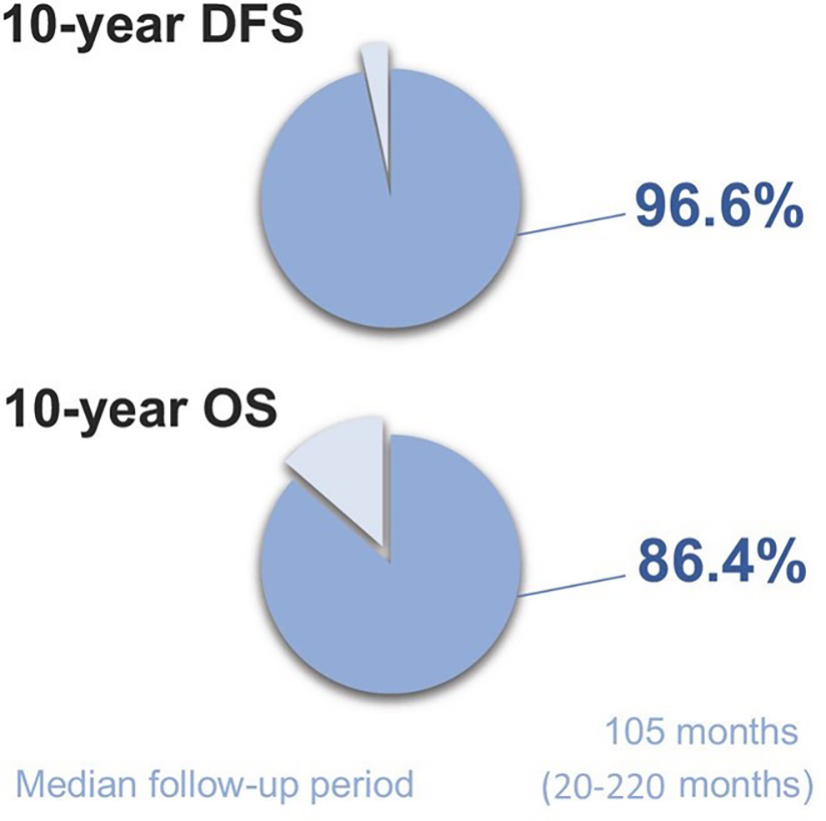
Summary of study findings.

During follow up, 5 patients underwent re-operation for suspected recurrence, 4 of whom had adjuvant radiation treatment. Of these, only 2 patients (3.4%) had pathologically confirmed recurrence while the remaining three were reported as fibrotic changes secondary to radiation. Disease-free survival at 10 years was found to be 96.6%. Eight patients died secondary to co-morbid conditions unrelated to thymoma. In a surgical thymoma series with different surgical approaches, published in 2003, 70 patients with Stage I and II thymomas were presented. Only 2 patients developed recurrence (2.8%) and a 5-year survival of 91% was reported. Authors claimed that the patients who died during the follow-up period were tumor free (12). These recurrence and survival rates correspond with the findings of our study, albeit with longer follow up period in our case (Figure 7). These favorable outcomes may be explained by the following: our study population does not include patients with thymic carcinoma, all resections were considered to be R0 (facilitated by use of frozen section and orientation of the specimen with the pathologist), high risk patients with invasion to adjacent organs were excluded from the study, and there was a high rate of adjuvant radiation therapy, in keeping with the recommendations at the time of the study.

Minimally invasive resection of Masaoka Stage I and II thymomas may be considered safe. However, in patients with clinical Masaoka Stage III disease, phrenic nerve, lung, pericardial, or pleural resections may be needed. Even, when there is no invasion into these structures, radiologically they may be misinterpreted especially in borderline Masaoka Stage II. Greater experience in minimally invasive techniques may be required to prevent unnecessary tissue resection and damage in higher stage patients (13).

Long term outcome studies with a robotic platform for thymoma resections generally have limited duration of follow-ups. Authors, with a propensity score-matched analysis, compared minimally invasive and open surgery for thymoma resections, were unable to find significant differences in margin positivity and 5-year survival (89.4% vs. 81.6%) (1). There has been no significant difference in the R0 resection rate and locoregional recurrence in patients with Masaoka Stages I and II tumors in a meta-analysis when minimally invasive and open thymoma resections were compared (14). A multi-institutional experience with robotic thymectomy in 131 patients demonstrated that, 97.8% of patients were reported alive with a median of 42 months (5–159 months). A pleural recurrence was the reason for recurrence in one (0.7%) patient. The five-year overall survival rates were 97%, and the five-year thymoma-related survival rates were 100% (2).

In a recently published article, the midterm outcomes of robotic thymoma surgery were reported to be exceptional (11). Disease specific survival of the patients with thymoma and thymic carcinoma was 100% and 95.2%, respectively (11). Out of 158 patients operated with robotic surgery, two patients were reported to die from non-thymoma related causes and one patient with thymic carcinoma. In this experience Median follow-up time was 43 months (11).

Minimally invasive surgery for thymoma has always been linked with possible higher potential risk for pleural dissemination, which could be attributed to opening of the capsule during the surgery. Larger and cystic thymoma have higher risk for pleural dissemination (15). Recurrence rates of thymoma have been reported after minimally invasive surgeries changing from 0% to 6.7%, with a reported 5-year disease-free survival ranging from 83.3% to 96% (8, 16–19) (Table 1). Surgical treatment for Masaoka Stage I and II thymoma, has been reported to have an overall 5-year survival rate of 89% to 100% and disease-free survival of 71% to 95%, respectively, regardless of the approach (20). Minimally invasive thymoma series provided similar outcomes (20).

**Table 1:** Patient demographics.

Age	Mean 47,62 (21–75)	
Gender	Female	32
	Male	27
Osserman-Genkins classification	Stage I	15 (25.5%)
	Stage IIA	22 (37.3%)
	Stage IIB	14 (23.7%)
	Stage IIIA	3 (5%)
	Stage IIIB	5 (8.5%)
Myasthenic medication		Preoperative prescription
	Pyridostigmine	54 (91.5%)
	Steroids	30 (50.8%)
	Azathioprine	5 (8.5%)
	IVIG	27 (45.7%)
Masaoka staging	Stage I	17 (28.8%)
	Stage II	42 (71.2%)
WHO classification	A-AB	18
	B1	14
	B2	22
	B3	5
Adjuvant RT	+	21 (35.6%)
	−	38 (64.4%)
Median stays (days)	ICU	1 (0–52)
	Hospital	4 (2–68)
Surgical technique	VATS	58
	RATS	1
Recurrences		Time to recurrence (months)
	Masaoka	
	Stage I	None
	Stage II	58th and 114th
	Osserman-Genkins	
	I	None
	II	58th and 114th
	III	None
	
	No adjuvant	114th
	Adjuvant	58th

We have also presented some of the MG data based on the medications patients were on. Our data suggests that most of our patients did better after the surgery, whereas some MG resistant patients (9 patients) underwent additional immunosuppressive therapies (Table 2). The neurological outcomes of MG patients after robotic surgery have also been studied in patients with a thymoma resection (13). In a series by Romano et al., patients who had their thymus removed showed a complete stable remission (CSR) rate of 14.7% at 3 years and a clinical improvement in 77% of patients with MG. Conversely, 23.6% experienced either no substantial change or worsened symptoms.

**Table 2:** Medication changes during the follow up.

	Preoperative treatment	Did not use postoperatively	Same dose as preoperative	Decreased the amount prescribed	Increased the amount prescribed	Started postoperatively
Pyridostigmine	54 (91.5%)	38 (70.4%)	4 (7.4%)	10 (18.5%)	2 (3.7%)	
Steroids	30 (50.8%)	22 (73.3%)	1 (3.3%)	6 (20%)	1 (3.3%)	2
Azathiopurine	5 (8.5%)	1 (20%)	4 (80%)			9
IVIG	27 (45.7%)					2

One last important point may be needed to stress here. The results of VATS and RATS resections in cancer patients depend on the surgeon's training and skills. The team started the VATS thymectomy surgery in 2002 and shared their experience in the following years (21, 22). This point is quite relevant to avoid newcomers to the specialty being involved in complex techniques before being trained simply because good results are published.

In conclusion, minimally invasive thymoma resections show similar outcomes in the long term follow up with conventional methods. This study has the longest median follow up of MG patients after thymoma resection, in the literature to date (Table 3).

**Table 3:** Minimally invasive thymoma resection and long term outcomes.

	Number of patients	Tumor size (mm)	Follow-up (Months)	5-year survival (%)	Recurrence (*N*)
Liu et al. (17)	Stage I: 57	46	44	96.9 (DFS)	2
	Stage II:19				
Ye et al. (18)	Stage I: 80	32.3	41	NA	1
	Stage II:45				
Sakamaki et al. (8)	Stage I: 40	35	48	92.4 (RFS)	2
	Stage II:31				
Odaka et al. (19)	Stage I: 33	40	55	95.8 (DFS)	2
	Stage II: 34				
Kang et al. (14)	Stage I:126	46	43	93.9 (RFS)	1
	Stage II:19				
Toker et al	Stage I:17	36	105	10 year 96% (RFS)	2
	Stage II:42				

## Data Availability

The raw data supporting the conclusions of this article will be made available by the authors, without undue reservation.
